# Autophagy and its role in regeneration and remodeling within invertebrate

**DOI:** 10.1186/s13578-020-00467-3

**Published:** 2020-09-21

**Authors:** Qian Song, Hongjin Liu, Hui Zhen, Bosheng Zhao

**Affiliations:** Laboratory of Developmental and Evolutionary Biology, Shandong University of Technology, Zibo, 255049 Shandong China

**Keywords:** Autophagy and regeneration, Autophagy-related genes (ATGs), mTOR, *Hydra vulgaris*, *Dugesia japonica*, Leech, *Hirudo medicinalis*

## Abstract

**Background:**

Acting as a cellular cleaner by packaging and transporting defective proteins and organelles to lysosomes for breakdown, autophagic process is involved in the regulation of cell remodeling after cell damage or cell death in both vertebrate and invertebrate. In human, limitations on the regenerative capacity of specific tissues and organs make it difficult to recover from diseases. Comprehensive understanding on its mechanism within invertebrate have strong potential provide helpful information for challenging these diseases.

**Method:**

In this study, recent findings on the autophagy function in three invertebrates including planarian, hydra and leech with remarkable regenerative ability were summarized. Furthermore, molecular phylogenetic analyses of DjATGs and HvATGs were performed on these three invertebrates compared to that of *Saccharomyces cerevisiae*, *Caenorhabditis elegans*, *Drosophila melanogaster*, *Mus musculus* and *Homo sapiens*.

**Results:**

In comparison with *Scerevisiae*, *C elegans*, *D melanogaster*, *M musculus* and human, our analysis exhibits the following characteristics of autophagy and its function in regeneration within invertebrate. Phylogenetical analysis of ATGs revealed that most autophagy-related genes (ATGs) were highly similar to their homologs in other species, which indicates that autophagy is a highly conservative biological function in both vertebrate and invertebrate. Structurally, almost all the core amino acids necessary for the function of ATG8 in mammal were observed in invertebrate HvATG8s and DjATG8s. For instance, ubiquitin-like domain as a signature structure in each ATG8, was observed in all ATG8s in three invertebrates. Basically, autophagy plays a key role in the regulation of regeneration in planarian. DjATG8-2 and DjATG8-3 associated with mTOR signaling pathway are sophisticated in the invertebrate tissue/organ regeneration. Furthermore, autophagy is involved in the pathway of neutralization of toxic molecules input from blood digestion in the leech.

**Conclusions:**

The recent investigations on autophagy in invertebrate including planarian, hydra and leech suggest that autophagy is evolutionally conserved from yeast to mammals. The fundamental role of its biological function in the invertebrate contributing to the regeneration and maintenance of cellular homeostasis in these three organisms could make tremendous information to confront life threatening diseases in human including cancers and cardiac disorders.

## Background

Autophagy is an evolutionarily conserved process, which plays a crucial role in maintaining cellular homeostasis by removing defective proteins, organelles and invading pathogens [1, 2]. Based on different mechanisms by which intracellular cargos are delivered to lysosomes, three forms of autophagy have been identified—chaperone-mediated autophagy (CMA), microautophagy and macroautophagy (the usual autophagy) [3, 4]. Multiple lines of evidence suggest that autophagic degradation is triggered by various stress responses, such as hypoxia [5], inflammation [6], and nutrient deficiency [7]. Due to its crucial role in maintaining cellular homeostasis, dysfunction of autophagyis thought to be associated with numerous diseases, including cancer, age-related disorders, infection, regeneration, et al. For example, in cancer, autophagy plays a dual role in different environments and tumor stages [8, 9]. In the early stage of tumorigenesis, autophagy acts as aninhibitor through its cellular qualitycontrol function, while in the late stage of tumorigenesis, autophagy provides a protective mechanism for maintaining cancer cell survival and homeostasis. According to Nilsson, deficient autophagy can disrupt the secretion of Aβ peptides, while the accumulated intracellular Aβ peptides can lead to Alzheimer’s disease (AD)-related pathology [10]. Moreover, autophagy-related genes (ATGs),such as ATG7, CDK5 and Beclin 1, may mediate the cross-talk between molecular mechanisms of autophagy and AD [11].

Regeneration is needed in maintaining homeostasis and adapting to the external environment due to apoptosis. Growing evidence has demonstrated that in mammals, autophagy is responsible for the repair of damaged tissues and the replacement of impaired organs or body parts after injury. For example, in muscle regeneration, autophagy may regulate proteostasis and survival mechanisms in regenerating fiber. Dysfunction of autophagy will lead to a decline in the function and number of muscle satellite cells, while restoration of autophagy can effectively prevent senescence and restore regenerative functions of geriatric satellite cells [12]. Additionally, autophagy plays a vital role in maintaining quiescence and stemness of cells by clearing active and healthy mitochondria in hematopoietic stem cells (HSCs) [13].

Regenerative ability may vary from species, organs, tissues, and even development stages [14]. In human, limitations on the regenerative capacity of specific tissues and organs make it difficult to recover from diseases. Compared with mammals, most invertebrates, such as planarian, hydra and leech, have remarkable abilities to regenerate any missing part after amputation [15–17]. A large population of adult stem cells may explain the astonishing regenerative abilities of planarians and hydras, while leeches, which have only a few stem cells, achieve their regeneration by dedifferentiation of tissue cells and migration and proliferation of stem cells [17]. Consistent with observations in vertebrates, autophagy appears to be a response to starvation as well as to injury in planarians and hydras [18, 19]. In starving animals, dramatic increase in the number of autophagic vacuoles was detected. An appropriate regulation of autophagy guarantees regeneration efficient in these invertebrates [19, 20]. In regenerating hydra, excessive autophagy induced by *Kazal1* silencing leads to death [21]. Treatment with rapamycin, a depressor of autophagy, delays the early phases of head regeneration in both fed and starved hydra. Besides, the autophagy inhibitors Wortmannin and Bafilomycin can also slightly delay head regeneration [19]. *Gtdap-1*, the planarian ortholog of human death-associated protein-1 (DAP-1), is involved in remodeling by a process of autophagy during planarian regeneration and starvation [18].

Investigating the cellular function of autophagy in regeneration process will allow us to know more about the situation in proliferation-related diseases and will contribute to the development of therapeutic strategies for human disorders. In comparison with vertebrates, invertebrates including planarian, hydra and leech present special characteristics that make them be valuable models to study the relationship between autophagy and regeneration: (1) in contrast to mammals where autophagy only occurs at specific times or in very specific organs, they offer unique models where autophagy occurs continuously due to their un-paralleled regenerative capability and continual process of change. (2) using them to study autophagy means addressing roles of autophagy in regeneration at a whole-organism level, but not at an organ level or asystem level [19, 22, 23]. Therefore, to further assess the role of autophagy in regeneration, ATGs and functional roles of autophagy in planarian, hydra and leech are mainly described in this article.

## ATG family and mTORC1-related remodeling within invertebrates

### ATG proteins involved in autophagy in general

Autophagy-related genes (ATGs) are essential for the formation of autophagosomes. Since the discovery of autophagy-related (ATG) genes initially in yeast, identification of ATG genes was undertaken in higher eukaryotes [24, 25]. Mammals contain almost all of them as well as a series of factors specific to higher eukaryotes.

Among these ATGs, one subset which is referred to as the “core” molecular machinery, plays a crucial role at different stages of autophagic process, i.e. initiation, elongation, maturation and fusion with lysosomes [3]. In mammals, these core ATG genes can be divided into several functional groups: (1) ULK1-ATG13-FIP200-ATG101 complex, (2) class III phosphatidylinositol 3-kinase (PtdIns3K) complex I, (3) two ubiquitin-like conjugation systems (ATG8/LC3 conjugation system and ATG12 conjugation system) and (4) ATG9 and its cycling system (ATG2, ATG9, ATG18) [26].

In mammals, initiation of autophagy occurs through ULK complex consisting of ULK1/2, ATG13, FIP200 and ATG101. ULK1/2, a homolog of yeast ATG1, contains an N-terminal kinase domain, a LIR motif and two C-terminal MIT domains [27]. Autophosphorylation of ULK1at Thr180 is crucial for activation [28]. MIT domain of ULK1 binds to MIM domain of ATG13, and ATG13 recruits ULK1 to FIP200 (a focal adhesion kinase family-interacting protein of 200 kDa). FIP200, a hybrid molecule of ATG17 and ATG11 [27], contains an N-terminal ATG17-like domain, a LIR motif, a coiled-coil region and a C-terminal Claw domain. Both ATG13 and FIP200 can stabilizeULK1/2 and increase its kinase activity [29, 30]. Besides MIM domain, ATG13 in mammals also contains an N-terminal HORMA and a LIR motif. The LIR domains of ULK1 and ATG13 in humans can mediate their interaction with ATG8s [31]. The ATG13 containing HORMA domain forms a heterodimer with ATG101 containing HORMA domain [32]. Therefore, the association of ATG101 with ATG13 is the key to autophagy induction [33]. Notably, ATG101 is an entirely novel ATG protein in mammals [34], contributing to maintaining the stability and basal phosphorylation of ATG13 and ULK1 [35, 36]. The WF-finger motif of ATG101 can recruit downstream proteins to the autophagosome formation site in mammals [37], and the C-terminal region is responsible for the binding of phosphatidylinositol 3-kinase (PtdIns3K) complex [32].

Class III PtdIns3K complex I, consisting of VPS34, VPS15, Beclin1 and ATG14(L)/Barkor, is a functional effector of ULK complex and contributes to promoting autophagy elongation [29]. VPS34, composed of an N-terminal lipid-binding C2 domain, a helical domain and a C-terminal kinase domain, is responsible for phosphorylating phosphatidylinositol and thus producing P13P [38]. VPS15 contains an N-terminal kinase domain, a HEAT domain and a C-terminal WD40 repeat domain. Beclin-1, a homology of ATG6, contains a coiled-coil domain and a BABA domain [39]. ATG14L is composed of a coil-coil domain and a BATs domain [27]. When ULK1 phosphorylates BECN1 on Ser14, the ATG14L-containing VPS34 complex is then activated. The cysteine-rich domain near the N-terminal of ATG14L plays a vital role in its starvation-induced translocation to the phagophore initiation sites [40]. BATs domain is required for ER localization of PI3KC3-C1, whereas the C-terminal region of VPS34 determines the orientation on the membrane [41].

In mammals, ATG8 protein is comprised of seven homologs: LC3A, LC3B, LC3C, LC3B2, GABARAP, GABARAP‐L1 and GABARAP‐L2/GATE‐16 [42]. All ATG8/LC3 proteins contain conserved C-terminal ubiquitin-like structures despite the lack of similarity in amino acid sequence [43]. The ubiquitin-like structure, comprising four β-strands and two α-helices, is responsible for the protein–protein interaction (PPI) [44]. The two amino-terminal α helices, which differ among ATG8 proteins, have their specific roles during autophagy. Emerging evidence suggests that LC3 mediates the elongation step, while GABARAP and GABARAPL2 are involved in the sealing and fusion of autophagosome [45]. Among four homologs (ATG4A, B, C, D) of the protease ATG4 in mammals, ATG4B, which is composed of a conserved papain-like domain and a unique short-finger domain according to the structural studies [27], plays a crucial role in processing all ATG8 family proteins [46]. In the process of autophagy, ATG8 is cleaved by ATG4at C-terminus to generate the cytosolic ATG8-1 with a glycine residue. Then, the glycine residue is covalently conjugated in a reaction catalyzed by ATG7/ATG3.

ATG7 is an E1-like enzyme that includes two domains, the N-terminal domain (ATG7-NTD) which can specifically recruit two distinct autophagic E2-like proteins, ATG3 and ATG10 [47], and the C-terminal domain (ATG7-CTD)which is involved in binding and activating ATG8 and ATG12 [27]. The ATG12 can be conjugated to ATG5 in a reaction catalyzed by ATG7 and ATG10. The ATG12-ATG5 conjugate can be directly recruited to phagophore by ATG16L in the interaction between noncovalently and ATG5 via a coiled-coiled domain [48]. The ATG12-ATG5-ATG16L complex can interact with ATG3 and facilitate the transfer of ATG8-like proteins from ATG3 to phosphatidyl ethanolamine (PE).

ATG9 is a six-transmembrane protein, the only known transmembrane protein in ATG core proteins, with both the N and C terminal in the cytosol. The function of ATG9 remains a mystery. In mammalian cells, ATG9 (called mATG9) resides in a unique endosomal-like compartment and on endosomes [49]. The mATG9 is required for the formation of phagophores and its trafficking to phagophore is regulated by TBC1D14 and TRAPPIII independent of early autophagy proteins, such as ULK1 [50]. And the fusion of ATG9 vesicles may provide the membrane structures for the growing phagophore [51].

### ATG family within invertebrates

Attention has been shifted from higher eukaryotes (e.g. yeast) to invertebrates in identifying the cellular basis of autophagy and the homologs of ATGs [52–54]. During evolution, ATGs have been duplicated and lost, thus resulting in the extinction and expansion of some subfamilies of autophagy-related genes. For instance, multiple ATG8 genes can be found in mammals, whereas there is only a single ATG8 gene in fungal species (e.g. yeast) [42]. Increasing number of yeast ATG orthologs were identified in *Hydra vulgaris* (*H. vulgaris*) and *Dugesia japonica* (*D. japonica*).

DjATGs include thirteen single genes and three ATG8 family-encoding genes (DjATG8-1, DjATG8-2, and DjATG8-3). Analysis of detailed biochemical index of these DjATG proteins showed their lengths ranged from 106 (DjATG12) to 1790 amino acids (DjATG2). The predicted molecular weights ranged from 11.9 kDa (DjATG12) to 205.9 kDa (DjATG2), pI ranged from 4.75 (DjATG3) to 9.16 (DjATG8-2), and gravity ranged from −0.644 (DjATG8-1) to 0.044 (DjATG9), suggesting that there were significant variations and potential functional differentiation. Based on sequence alignment, DjATGs could be divided into two groups: group with high identity and group with low identity. The former group includes DjATG3, DjATG4, DjATG5, DjATG7, DjATG8 and DjATG12 (> 35%), while the rest falls into the latter group (Table1).

**Table 1:** ATG proteins in *Dugesia japonica* and *Hydra vulgaris*

Protein complexes	Proteins	Gene accession	Locus name	AA	PI	Mw (kD)	GRAVY	Identity (%)					Functions and characteristics	References
								*Hs*	*Mm*	*Dm*	*Ce*	*Sc*		
*ATG1 complex*													*Initiation of autophagy*	
	ATG1	AWD06772.1	DjATG1	814	7.23	92.0	−0.451	26	26.4	26.4	27.4	18.2	Serine/threonine kinase	[24, 51–53]
	ATG13	AWD06777.1	DjATG13	401	5.38	45.1	−0.504	21.6	21.3	18.1	18.1	11.9	Phosphoprotein	[25, 26, 29, 54, 55]
		CDG71824.1	HvATG13	432	5.23	49.1	−0.270	23.5	23	24.5	16.7	13.6		
	ATG101	CDG67707.1	HvATG101	230	6.44	27.1	−0.505	48.2	48.2	43.6	–	–	Link the ATG1/13 complex to autophagic membranes	[31, 32]
*PI3K complex*													*Autophagosome formation*	
	ATG6	CDG70076.1	HvBECN1	451	5.07	52.2	−0.672	60	60.4	53.7	28	26.8	Allosteric modulator of PI3KC	[56–58]
		AVX32557.1	DjATG6	423	4.94	49.0	−0.480	33.7	33.5	33.8	25.4	16.9		
	ATG14	CDG69295.1	HvATG14	467	7.05	53.6	−0.487	25.7	26.2	22.6	10	12.6	Regulates autophagosome targeting	[36, 37, 59–62]
*Ubiquitin-like conjugation (ATG12)*													*Conjugation of ATG12 and ATG5*	
	ATG5	CDG67424.1	HvATG5	285	7.78	33.8	−0.428	56.3	55.9	45.1	33.6	21.4	Target for ATG12 conjugation	[63–66]
		AWD06774.1	DjATG5	284	5.81	33.2	−0.263	41	40.7	41.5	32.4	21.3		
	ATG7	CDG71639.1	HvATG7	693	5.39	78.2	−0.126	52.5	53.7	42.8	–	41.5	E1 conjugation enzymes for ATG12 conjugation	[43, 67–69]
		APY27057.2	DjATG7	693	6.39	79.1	−0.267	39.7	40.4	39.1	–	36.8		
	ATG10	CDG67441.1	HvATG10	192	5.65	22.5	−0.193	35.4	34.4	30.1	26.5	18.8	E2 conjugation enzymes for ATG12 conjugation	[70, 71]
		AWD06776.1	DjATG10	165	6.42	19.5	−0.284	23.8	23.8	23.4	19.6	16		
	ATG12	CDG71705.1	HvATG12	129	5.44	14.7	−0.508	48.8	47.3	50	31	18.3	Ubiquitin−like modifier, ATG5 and ATG10 interaction	[44, 63–66, 70]
		AVL25105.1	DjATG12	106	5.59	11.9	−0.187	45.7	46.7	39.4	37.7	28.4		
	ATG16	CDG69520.1	HvATG16L1	491	6.95	55.5	−0.458	32.6–43.6	32–43.6	42	29.7–30.8	10.6	Required for the localization of ATG5–ATG12 to membranes	[47, 72, 73]
		AWD06778.1	DjATG16	529	8.1	60.2	−0.500	28.1–34.2	27.5–34.2	33	24.9–27	9.7		
*Ubiquitin-like conjugation (ATG8)*													*Conjugation of ATG8 to PE*	
	ATG3	CDG67081.1	HvATG3	308	4.79	35.2	−0.614	60.5	67	61	52.2	36.7	E2 conjugation enzymes for ATG8 conjugation	[64, 74–80]
		ASL04728.1	DjATG3	322	4.75	36.8	−0.457	51.1	56.9	55.8	48	33		
	ATG4	CDG66347.1	HvATG4B	560	6.44	64.2	−0.209	26.5–47.6	25.8–46.8	28.2–39	25.8–36.4	21	Cysteine protease	[81–88]
		CDG68148.1	HvATG4C	442	7.13	51.0	−0.418	29.4–39.7	27.9–39.7	29.8–37.7	26.9–27.8	23.8		
		AQK38494.1	DjATG4	412	5.84	47.1	−0.251	29.3–42.7	29.7–43.3	30.2–38	26.8–34	25.9		
	ATG7	CDG71639.1	HvATG7	693	5.39	78.2	−0.126	52.5	53.7	42.8	–	41.5	E1 conjugation enzymes for ATG8 conjugation	[64, 69, 70, 89–91]
		APY27057.2	DjATG7	693	6.39	79.1	−0.267	39.7	40.4	39.1	–	36.8		
	ATG8	CDG71662.1	HvGABARAP	118	7.92	14.1	−0.607	30.2–94.9	30.2–94.9	75.4–89	33.6–82.2	54.7	Ubiquitin-like modifier, recruitment and scaffolding of proteins, cargo recognition	[38, 39, 41, 92–97]
		CDG70632.1	HvGABARAPL2	118	8.66	13.7	−0.340	39.3–71.8	37.6–71.8	55.9–63.6	37.9–62.7	62.4		
		XP_012555909.1	HvLC3A	129	9.18	15.2	−0.410	40.3–52.9	41.9–52.9	39.7–39.8	39.7–42.7	37.6		
		CDG67574.1	HvLC3C	125	9.45	14.5	−0.510	37.6–70.4	37.6–62.8	38.5–40.2	41.9	39.3		
		APU52177.1	DjATG8-1	117	7.89	14	−0.644	32.5–86.3	32.5–86.3	76.9–85.5	33.6–82.1	53.8		
		APU52176.1	DjATG8-2	119	9.16	13.9	−0.360	35.3–66.7	36–66.7	50.0–50.4	38.8–51.3	47.9		
		APU52178.1	DjATG8-3	118	6.74	13.8	−0.511	34.8–59.8	33.9–59.8	55.1–61.0	31.9–56.8	77.8		
*ATG9 complex*													*Membrane recruitment to autophagosomes*	
	ATG2	CDG68195.1	HvATG2	1296	5.13	145.5	−0.294	34.7–36.7	34.9–35.2	27.4	20.5	14.6	Transfer lipids and bridges the forming autophagosome to ER	[98–106]
		AWD06773.1	DjATG2	1790	5.41	205.9	−0.367	16.8–18	16.7–18	17.4	15.5	12		
	ATG9	CDG69175.1	HvATG9	790	8.67	91.9	−0.082	32.1–41.5	33–41.4	33.1	27.3	21.8	Integral membrane protein	[102, 107–114]
		AWD06775.1	DjATG9	741	8.42	85.5	0.044	23.6–26.2	23.4–26.1	26.2	23.1	19.3		
	ATG18	AWL25033.1	DjATG18	446	6.79	50.1	−0.162	24.9–33.7	24.3–34.5	30.9–31.6	33.5	29.8	Function for ATG2 localization	[99, 101, 115]

AA, amino acids; Hs, *Homo sapiens*; Mm, *Mus musculus*; Dm, *Drosophila melanogaster*; Ce, *Caenorhabditis elegans*; Sc, *Saccharomyces cerevisiae*

**Fig. 1:**
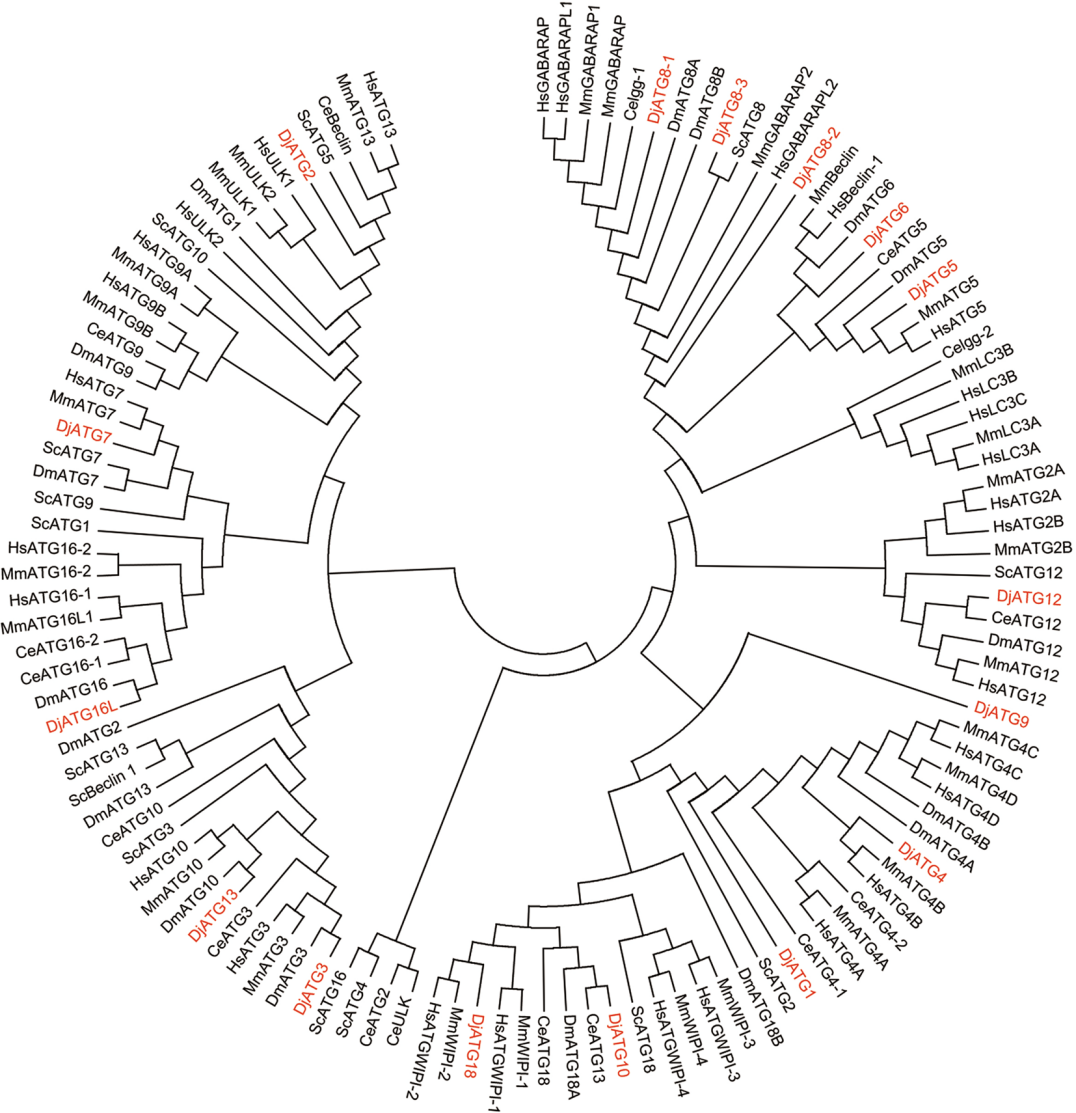
Molecular phylogenetic analysis of ATGs by Maximum Likelihood. The evolutionary tree is presented to compare each subgroup with family members present in other species. Bootstrap analysis was performed with 1000 replicates. Evolutionary analyses were conducted in MEGA-X. The proteins were analyzed as intact sequences. The analysis involved genes from *D. japonica* (Dj), *S. cerevisiae* (Sc), *C. elegans* (Ce), *D. melanogaster* (Dm), *M. musculus* (Mm), and *H. sapiens* (Hs). The names in red color are the *D. japonica* ATGs

ATG protein sequences of*Homo sapiens* (*H. sapiens*), *Mus musculus* (*M. musculus*), *Drosophila melanogaster* (*D. melanogaster*), *Caenorhabditis elegans* (*C. elegans*) and *Saccharomyces cerevisiae* (*S. cerevisiae*) were collected and aligned with those of *D. japonica*. Phylogenetically, some gene families were highly similar to their homologs in other species (Fig. 1). For instance, ATG5, ATG8 and ATG12 of six species were clustered together, suggesting that they were evolutionally conserved and might have originated from a common ancestor. However, the separation of ATG1, ATG2, ATG9, ATG10 and ATG13 by other ATGs indicated a relatively high variation in protein sequences.

**Fig. 2:**
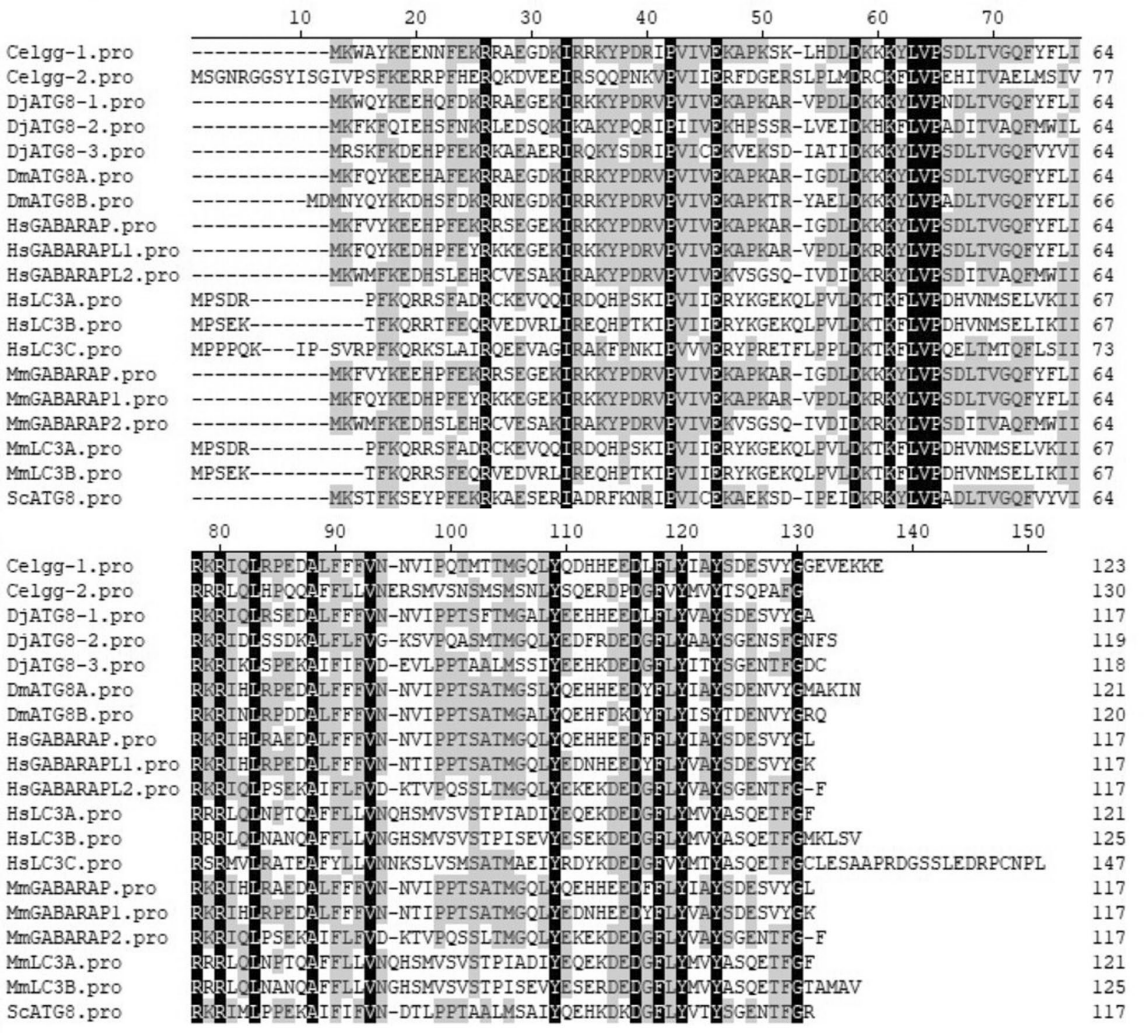
Multi-alignment analysis of ATG8 family proteins. Black shading indicates position with fully conserved redsidues. *D. japonica* (Dj), *S. cerevisiae* (Sc), *C. elegans* (Ce), *D. melanogaster* (Dm), *M. musculus* (Mm), and *H. sapiens* (Hs)

Compared to the single ATG8 gene present in yeast, there are three ATG8 orthologues present in *D. japonica*. Sequence alignment of ATG8s displayed 20 amino acids with conserved sequences in all proteins (black), indicating a highly conserved primary amino acid sequence (Fig. 2). ATG8-interacting motif (AIM) interacts with two adjacent hydrophobic pockets (HP1 and HP2) of ATG8, with HP1 composed of Glu^17^, Ile^21^, Pro^30^, Ile^32^, Lys^48^ and Leu^50^, and HP2 composed of Tyr^49^, Val^51^, Pro^52^, Leu^55^, Phe^60^ and Val^63^ [79]. Under the interaction of ATG8 and ATG3, Val^31^, Lys^46^, Lys^48^, Tyr^49^, Leu^50^, Val^51^, Val^63^ and Ile^64^ play crucial roles. Besides, new evidence has indicated that Arg^65^, Phe^104^ and Tyr^106^ in yeast ATG8 contribute to the conjugation of ATG8 to PE and the C-terminal glycine [120]. Results suggested that almost all the core amino acids, except Ile^32^, Tyr^49^, Leu^55^, Phe^60^ and Val^63^, are necessary for the function of ATG8 were observed in all proteins. Notably, in *D. japonica*, a mutant of Val^31^ was observed. Besides, the 6th and 22nd amino acids in DjATG8-2 in *D. japonica* are glutamine and lysine respectively; but in other proteins, they are lysine and arginine.

Genomic DNA of Hydra Vulgaris encodes six ATGs from HvATG4 and HvATG8 gene families, and others encoded by a single gene. HvATGs were composed of 118 (HvGABARAP and HvGABARAPL2) to 1296 amino acids (HvATG2), with corresponding molecular weights from 13.7 kDa (HvGABARAPL2) to 145.5 kDa (HvATG2), pI from 4.79 (HvATG3) to 9.45 (HvLC3C), and gravity from -0.672 (HvBCEN1) to -0.082 (HvATG9). Based on sequence alignment, most HvATGs, including HvATG4, HvATG5, HvBECN1, HvATG9, HvATG10, HvATG12 and HvATGATG16L1, were highly similar to those of mammals, while HvATG3, HvATG7, HvATG8s and HvATG101 share high identity with other species (> 35%) (Table 1).

**Fig. 3:**
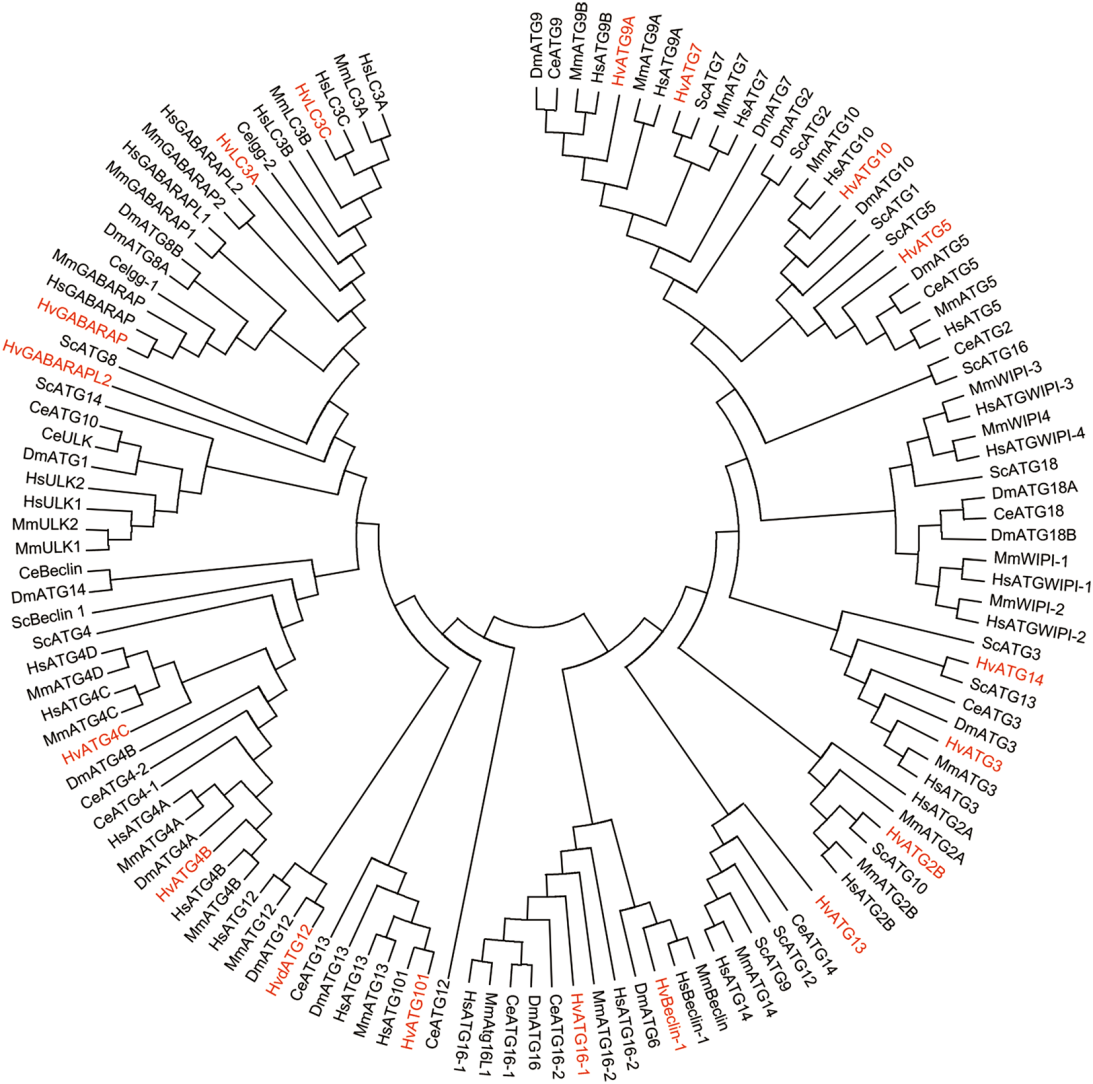
Phylogenetic analysis of ATG family proteins by Maximum Likelihood. The evolutionary tree is presented to compare each subgroup with family members present in other species. Bootstrap analysis was performed with 1000 replicates. Evolutionary analyses were conducted in MEGA-X. The proteins were analyzed as intact sequences. Phylogenetic relationships of ATGs from *H. vulgaris* (Hv), *S. cerevisiae* (Sc), *C. elegans* (Ce), *D. melanogaster* (Dm), *M. musculus* (Mm), and *H. sapiens* (Hs). The names in red color are the *H. vulgaris* ATGs

**Fig. 4:**
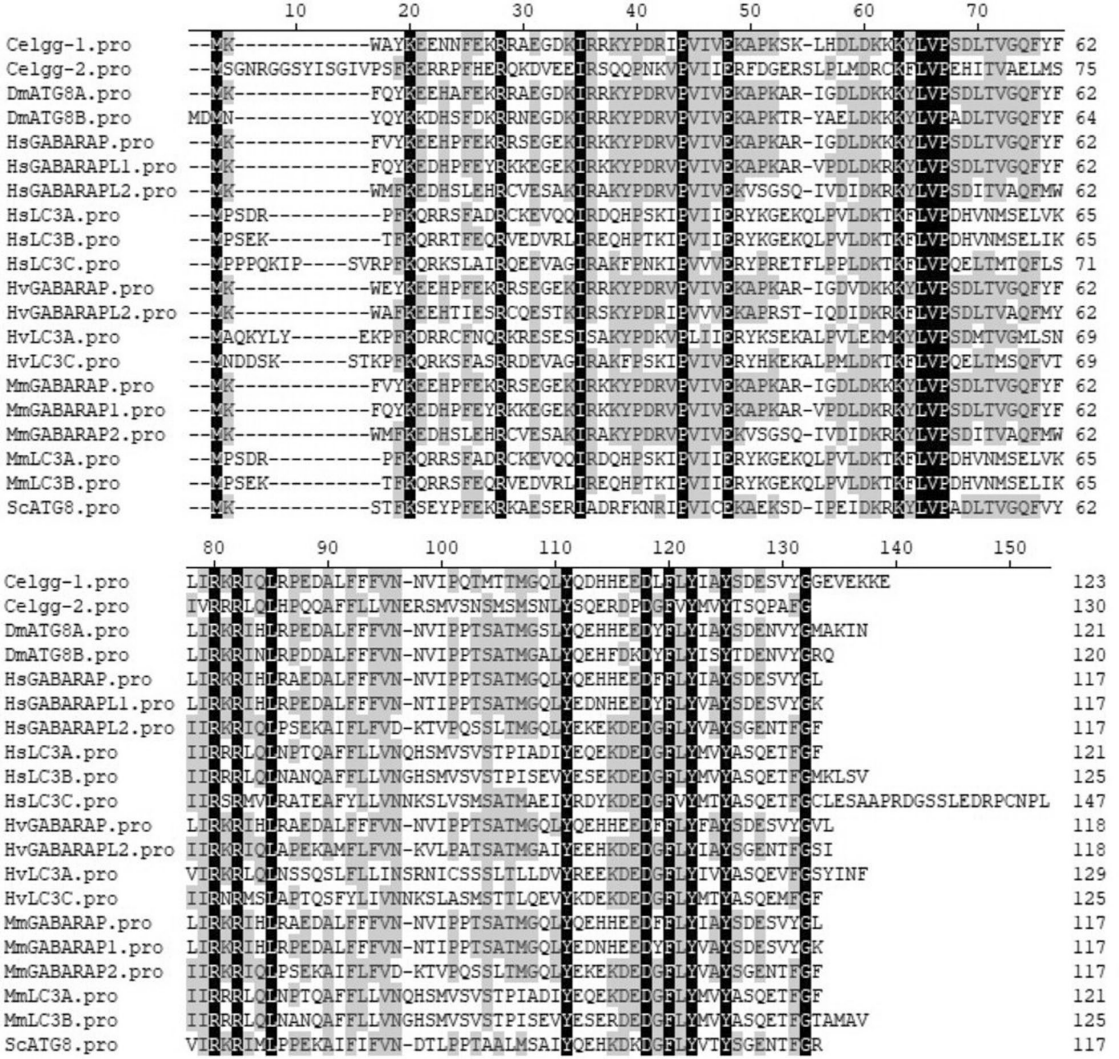
Multi-alignment analysis of ATG8 family proteins. Black shading indicates conserved amino acids. *H. vulgaris* (Hv), *S. cerevisiae* (Sc), *C. elegans* (Ce), *D. melanogaster* (Dm), *M. musculus* (Mm), and *H. sapiens* (Hs)

Molecular phylogenetic analysis of ATG proteins revealed that most HvATGs, except HvATG13 and HvATG14, were highly similar to their homologs in other species, indicating that ATGs in *H. vulgaris*, *H. sapiens*, *M. musculus*, *D. melanogaster*, *C. elegans* and *S. cerevisiae* had a common ancestor (Fig. 3). The sequence alignment of HvATG8s with other species indicated that HvATG8s had highly conserved primary amino acid sequences. Sequence alignment of ATG8s displayed 19 amino acids with conserved sequences in all proteins (black), including the core amino acids described above. Interestingly, the 40th amino acid in HvATG8is valine, while in other ATG8s, it is iso-leucine. The ubiquitin-like domain, a signature structure in each ATG8, was composed of 103–115 amino acids, as shown in Table 2 (Fig. 4).

**Table 2:** ATG8 family members in *Dugesia japonica* and *Hydra vulgaris*

Gene Name	NCBI	Ubiquitin-like domain (AA)	Region (AA)	Transcripts (bp)	CDS (bp)
DjATG8-1	APU52177.1	107	5–111	1014	354
DjATG8-2	APU52176.1	112	5–116	607	360
DjATG8-3	APU52178.1	103	11–113	1169	357
HvGABARAP	CDG71662.1	115	2–116	667	357
HvGABARAPL2	CDG70632.1	112	5–116	663	357
HvLC3A	XP_012555909.1	105	19–123	643	390
HvLC3C	CDG67574.1	113	11–123	934	378

### DjATG8 family contributes to tissue remodeling after amputation

A number of evidences have suggested the impact of autophagy during regeneration. For instance, induced autophagy in mice can increase microtubule stability through the degradation of SCG10, an MT-destabilization protein, thus promoting axon regeneration after injury [121]. A recent study showed that in a hypomorphic *ATG16L1* mouse with autophagy attenuated but still present, the recovery of skeletal muscle following cardiotoxin mediated damage was slower [122]. Autophagy also plays an important role in maintaining the proliferation of intestinal stem cells of fruit fly during aging and regeneration [123].

Using Planarians as an in vivo autophagy model, many studies carry on their experiments on the animal for remarkable plasticity and regenerating process. A study on *D. japonica* showed that DjATG8-2 (a homolog of *Schistosoma haematobium* GABARAPL2) and DjATG8-3 (a homolog of yeast ATG8) are involved in the tissue remodeling of planarians during regeneration [20]. Both DjATG8 proteins contain conserved ATG8 domains and three conserved amino acid residues (Arg65, Phe104 and Tyr106), which are essential for the conjugation of ATG8 to PE and C-terminal glycine; DjATG8-3 has similar structures in yeast ATG8 protein, with AIM peptide sites buried in two distinct pockets (W and L). The formation of autophagosomes is inhibited when expression levels of DjATG8-2and DjATG8-3 are down-regulated by RNAi. Then, both DjATG8-2and DjATG8-3 are expressed in blastema by WISH. During regeneration, up-regulation of expression levels of DjATG8-2 and DjATG8-3 is observed. However, the regeneration will be slowed down due to RNA interference of DjATG8-2 or DjATG8-3,and the loss of DjATG8-3will induce death after amputation and karyolysis in nucleus of planarian. In conclusion, the study of Kang et al. indicated that DjATG8-2 and DjATG8-3 play an essential role in the tissue remodeling of planarians during regeneration.

### mTOR signaling pathway associated autophagy in remodeling and regeneration

Mechanistic target-of-rapamycin (mTOR), a serine/threonine kinase, involves two functional complexes: mTORC1 and mTORC2. mTORC1, as a central regulator in cell metabolism and proliferation, is composed of mTOR catalytic subunit, Raptor, mLST8 and two inhibitory subunits (PRAS40 and DEPTOR) [27]. FKBP12-rapamycin complex binds to FKBP12-rapamycin-binding (FRB) domain, inhibiting the kinase activity of mTOR [124]. Tuberous sclerosis (TSC) tumor suppressor complex (TSC1/TSC2) indirectly inhibits mTORC1 activity by negatively regulating the activity of Rheb via the GTPase-activating protein (GAP) activity of TSC2 [125]. Activation of growth factor/PI3K/AKT signaling pathway, ERK1/2, and p90 ribosomal S6 kinase (RSK1) can inactivate TSC1/TSC2 complex, leading to the activation of mTOR [126–128]. In contrast, AMPK phosphorylates TSC2, resulting in the inhibition of mTORC1 activity [129].

In growing cells, autophagy is negatively regulated by high mTORC1 activity rather thanmTORC2. For instance, mTORC1 inhibits autophagy through direct phosphorylation ULK1 at the Ser758 site to prevent the interaction between ULK1 and AMPK, which is crucial for ULK1 activation [130]. mTORC1 can also prevent the formation of autophagosome through phosphorylation of ATG14L in VPS34 complex [131]. The prevention of nuclear translocation of transcription factor E3 (TFE3) and microphthalmia-associated transcription factor (MITF) by mTOR1 can provide an autophagy inhibition mechanism at the transcriptional level [132, 133]. Besides, accumulating evidence suggests that autophagy can also be regulated by acetylation. Wan et al. found that the phosphorylation of histone acetyl-transferase (HAT) p300 by mTOR leads to suppression of starvation-induced autophagy [134].

More studies have shown that mTOR is one of the critical regulatory signaling pathways of tissue regeneration in vertebrates and invertebrates. In mammalian cells, mTOR plays a different or even opposing role in diverse neuronal injury models. It’s reported that the mTOR signaling pathway differently regulates central and peripheral axon regeneration in mice [135]. Inhibition of mTOR by rapamycin dramatically can diminish the axon regeneration from embryonic cortical neurons. In contrast, mTOR is not required for adult DRG axonal regenerative ability. However, injury-induced neuronal mTOR activity boosts Stat3 signaling in PNS neurons, contributing to axon regeneration [136]. Moreover, the treatment of injured sciatic nerve of a rat with rapamycin, in which autophagy is induced by inhibiting the activation of mTOR, promotes the nerve regeneration and rebuilds the motor function [137]. Additionally, the overexpression of mutant HDAC5^AA^ in rats can result in an increase in HDAC5 cytoplasmic localization and activate the mTOR pathway, thus enhancing the regeneration ability of RGCs after optic nerve injury [138]. mTOR is also an important regulator for muscle regeneration. Peroxisome proliferator activated receptors γ (PPARγ) can be stimulated with nutmeg, which may be involved in myogenesis process of cardiac muscle. In aging rats, treatment with nutmeg may induce AKT-mTOR-autophagy pathway, thus increasing the muscle mass [139].

In *D. melanogaster*, TOR is required for the proliferation, growth and survival of germline stem cells (GSCs). When exposed to ionizing radiation, foxo paused the cell cycle of the damaged stem cells. TOR was able to overcome the action of foxo, and the stem cells resumed dividing and regenerating the damaged tissue [140]. What’s more, TOR activation in *D. melanogaster* intestinal stem cells (ISCs) is required for the rapid activation of ISC proliferation in response to a challenge [141].

Rapamycin that acts as a negative regulator of mTOR, efficiently induces autophagy in both intact and regenerating hydra. The transiently excessive autophagy might delay the early phase of head regeneration. During head regeneration, mTOR expression remains constant in the early phase of regeneration, progressively decreases in the early-late phase of regeneration and is finally dramatically up-regulated in the late phase of regeneration. It suggests that autophagy might participate in head regeneration at the early and early-late stages when mTOR is low, but inhibited at the late stage of regeneration [19]. A special hydra species named *H. oligactis* (*Ho*) undergoes aging when the temperature drops to 10 °C. Induction of an efficient autophagy is able to rescue epithelial cell cycling. However, in aging animals, rapamycin treatment restores epithelial proliferation but does not rescue the autophagy flux, suggesting that the positive effects are regulated by a distinct mechanism [142].

The role of mTOR signaling pathway in regeneration has also been identified in planarians. In *Schmidtea mediterranea* (*S. mediterranea*), inhibition of mTOR with RNA interference disrupts the behavior of neoblasts at the systemic level and severely restricts cell proliferation [143]. Emerging evidence has shown that mTOR signaling acts antagonistically with *Smed-smg-1* (a homolog of PIKK). *Smed-smg-1* (*RNAi*) results in a hyper-responsiveness to injury. Regenerative blastemas remain undifferentiated leading to lethal ectopic outgrowth*.* Loss of mTORC1 (*Smed-tor RNAi* or *Smed-raptor RNAi*) is capable of reversing the effects of *Smed-smg-1* (*RNAi*) by decreasing proliferation [144]. Rapamycin treatment can also prevent the tissue homeostasis and regeneration defects observed in *Smed-PTENRNAi* worms [145]. Besides, mTOR down-regulation leads to elongation of telomeres in planarian stem cells [146].

mTOR is reported to be involved in the regulation of regeneration in *D. japonica*, which is consistent with its role in *S. mediterranea* [147]. During regeneration, the expression level of DjTOR in posterior blastemas (PBs) surrounding the wound is up-regulated. Notably, the inhibition of DjTORwill lead to asymmetric blastemas and remarkable reduction growth, while rapamycin can successfully inhibit DjTORand induce autophagyin *D. japonica*. Therefore, worms treated with rapamycin displayed asymmetric blastemas and neuronal defects. In conclusion, DjTOR is involved in the regulation of regeneration in *D. japonica*.

## Bloodstream infection and autophagy via leech

Leeches are well-known for their blood-feeding habits and their extensive use in many human diseases. In relief of venous congestion and plastic and reconstructive surgery [148, 149], the efficient lysis and catabolism of blood can provide an abundance of nutrients for leeches. However, the degradation of hemoglobin, the most abundant protein in vertebrate blood, results in the generation of amino acids and heme, which may be toxic or even lethal [150, 151]. For example, under laboratory breeding conditions, signs of death of cells or even organisms given blood meals were observed [152, 153]. In order to maintain homeostasis, several mechanisms have been developed to neutralize toxic molecules in blood-feeding animals [154, 155]. It is reported that in *Ae. Aegypti*given blood meals, expression level of autophagy-related genes significantly increases [156]. Autophagy has also been shown to be a survival factor and involved in protecting epithelial cells from the toxic molecules caused by blood degradation in leeches [153].

In the previous studies, numerous vesicles with an electron-dense content in cytoplasm of midgut cells in *Piscicola geometra* were observed. They were originally described to be involved in the enzyme accumulation [157]. However, further study showed that the electron-dense content is formed by residual bodies of autolysosomes [153]. It was observed that autophagy occurred in all regions of digestive system (esophagus, crop, posterior crop caecum, and intestine) in adult non-feeding and feeding specimens. During autophagy, the autophagosomes engulfing the damaged organelles fused with lysosomes to form autolysosomes. Then cell membrane was disrupted by the accumulation of autophagosomes, autolysosomes or residual bodies, releasing autophagosomes, autolysosomes or residual bodies into midgut lumen. In digestive cells, autophagy occurred only in about 10–30% of cells before blood feeding, and was significantly up-regulated during and after bloodfeeding, compared with juvenile and non-feeding specimens, in which the process was absent. This suggests that autophagy is involved in the neutralization of toxic molecules caused by blood digestion in midgut epithelium of adult leeches.

## Conclusion

The identification ofautophagic process and a number of orthologs of ATGs in planarian, hydra and leech suggest that autophagy is evolutionarily conserved from yeast to mammals. Phylogenetical analysis of ATG proteins suggests that ATG proteins involved in ATG8 and ATG12 ubiquitin-like conjugation systems share high identity with their homologs, indicating that they might originate from a common ancestor. Distant homologs of ATG proteins were also found in both planarian and hydra, suggesting that they might have different functions. Notably, compared to *D. melanogaster*, *C. elegans* and *S. cerevisiae*, HvATGs show a higher identity with *H. sapiens* and *M. musculus*, suggesting that hydra can be used as a powerful model for uncovering the role of autophagy in human diseases.

Understanding the mechanisms of regenerative process has a clinical interest due to its effectiveness in many treatments for tissue repair and age-related diseases. Autophagy is strongly activated not only in starving planarians and hydras but also during regeneration. In leeches, autophagy is involved in the neutralization of toxic molecules caused by blood digestion. The results discussed above suggest that autophagy also plays a role in these three organisms when it can contribute to the regeneration and maintenance of cellular homeostasis. However, the control mechanisms of autophagy remain unclear, and the analysis of the relationship between autophagy and regeneration will provide a more comprehensive view of therapeutic strategies for human diseases.

## Data Availability

All data generated or analyzed during this study are included in this published article.

## Abbreviations

AA: Amino acids
AIM: Autophagy interacting motifs
AMPK: AMP-activated protein kinase
ATG: Autophagy-related gene
CDK 5: Cyclin Dependent Kinase 5
CTD: C-terminal domain
CTX: Cardiotoxin
DRG: Dorsal root ganglion
ER: Endoplasmic reticulum
ERK: Extracellular regulated protein kinases
FIP200: Focal adhesion kinase (FAK) family interacting protein of 200 kDa
FRB: FKBP12-rapamycin binding
GABARAP: γ‐Aminobutyric acid receptor‐associated protein
GATE-16: Golgi-associated ATPase enhancer of 16 kDa
HAT: Histone acetyltransferase
LC3: Microtubule‐associated protein light chain three
LIR: LC3-interacting region
MITF: Microphthalmia-associated transcription factor
Mm: *Mus musculus*
mTORC: Mechanistic target of rapamycin complex
Mw: Molecular weight
NTD: N-terminal domain
PE: Phosphatidylethanolamine
PNS: Peripheral nervous system
PPARγ: Peroxisome proliferator activated receptors γ
PtdIns3K: Phosphatidylinositol 3-kinase
RSK1: Ribosomal S6 kinase
TFE3: Transcription factor E3
mTOR: Mammalian target of rapamycin
TSC: Tuberous sclerosis
ULK: Unc-51-like kinase
VPS: Vacuolar protein sorting
WD: Tryptophan-aspartate
